# National Registry of Interstitial Lung Disease from Pakistan

**DOI:** 10.7759/cureus.14684

**Published:** 2021-04-25

**Authors:** Ali Bin Sarwar Zubairi, Mosavir Ansarie, Talha Mahmud, Saadia Ashraf, Nisar Ahmed Rao, Arshad Javaid, Zubair Shaheen, Shereen Khan, Afzaalullah Khan

**Affiliations:** 1 Medicine, Aga Khan University Hospital, Karachi, PAK; 2 Pulmonology, Doctors Plaza, Karachi, PAK; 3 Pulmonology, Sheikh Zayed Hospital, Lahore, PAK; 4 Pulmonology, Khyber Teaching Hospital, Peshawar, PAK; 5 Pulmonology, Dow University of Health Sciences, Karachi, PAK; 6 Pulmonology, Lady Reading Hospital, Peshawar, PAK; 7 Pulmonology, Nishtar Medical College Hospital, Multan, PAK; 8 Pulmonary and Critical Care, Bolan University of Medical & Health Sciences, Quetta, PAK; 9 Pulmonology, Gulab Devi Chest Hospital, Lahore, PAK

**Keywords:** interstitial lung disease, lung fibrosis, pulmonary fibrosis, rare lung diseases

## Abstract

Introduction

Interstitial lung disease (ILD) is a heterogeneous group of over 200 parenchymal lung diseases with a myriad of etiologies. Interstitial lung disease registries from around the world show varying prevalence and incidence of these diseases. The aim of this study was to determine the epidemiology and characteristics of ILD in Pakistan.

Methods

This web-based registry, which is the first multicenter registry of ILD from Pakistan, recruited patients from 10 centers of five major cities between January 2016 and March 2019.

Results

A total of 744 patients were enrolled in the registry. The five most frequent ILDs were idiopathic pulmonary fibrosis (IPF) 34.4%, hypersensitivity pneumonitis (HP) - 17.7%, idiopathic nonspecific interstitial pneumonitis (iNSIP) - 16.8%, connective tissue disease-associated ILD (CTD-ILD) - 16.3%, and sarcoidosis - 9.1%.

Conclusion

Idiopathic pulmonary fibrosis is the most prevalent ILD in Pakistan, followed by HP and iNSIP. An ongoing prospective registry with longitudinal follow-up will help us further elaborate on the clinical characteristics, treatment, and survival outcome of patients with ILD.

## Introduction

The term interstitial lung disease (ILD) encompasses a group of heterogeneous diseases that affect the lung parenchyma. The etiology of the disease is diverse. Two thirds of all ILD are idiopathic, most common of these is idiopathic pulmonary fibrosis (IPF) and idiopathic non-specific interstitial pneumonitis (iNSIP). Other causes of ILD may be secondary to environmental and occupational exposures as in hypersensitivity pneumonitis (HP) or as a manifestation of connective tissue disease (CTD) [1]. The diagnosis of ILD is often complex and requires a multi-disciplinary approach involving a team of pulmonologists, radiologists, pathologists, and rheumatologists in selected cases.

Although several worldwide epidemiologic studies on ILD have been carried out, the data from these studies is variable, partially dependent on the diagnostic criteria used [2-4]. Additionally, differences in geographic and ethnic populations may also play a role. Most epidemiologic studies on ILD have been conducted in Western countries, although data from the Eastern hemisphere is on the rise.

Serially published data between 2008 and 2016 on the relative frequencies of ILD limited to few centers from Pakistan suggests that 30 to 40 percent of reported ILD cases were IPF [5-9]. The second recently published report derives its data from the ongoing prospective national registry from multiple geographical locations of Pakistan documenting the epidemiology and characteristics of ILD [10]. This study includes the updated data of further three months subsequent to this report. The aim of this study was to describe the epidemiology and characteristics of ILD using the first multicenter web-based registry of Pakistan.

This article was previously presented as an abstract at the CHEST Annual Meeting on October 18-21, 2020.

## Materials and methods

**Figure 1:**
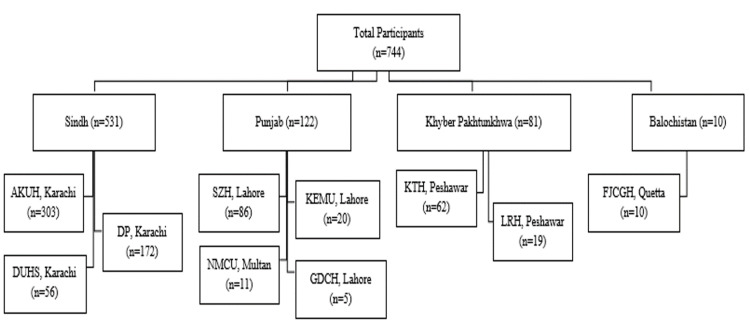
Recruitment from Different Centres of Pakistan AKUH=Aga Khan University Hospital, DP=Doctors Plaza, DUHS=Dow University of Health Sciences, SZH=Sheikh Zayed Hospital, KEMU=King Edward Medical University, NMCU=Nishtar Medical College, GDCH=Gulab Devi Chest Hospital, KTH=Khyber Teaching Hospital, LRH=Lady Reading Hospital, FJCGH=Fatema Jinnah Chest & General Hospital

In this prospective national registry under the supervision of the Pakistan Chest Society (PCS), recruitment of participants was done from January 2016 till March 2019. A total of 10 centers from five major cities across Pakistan participated in the registry (Figure 1).

The Independent Ethics Committee Approval (Ref. No. MU/ECA/99/861/R-2) was obtained for all participating centers and they were requested to assign a principal investigator (PI) who was responsible for patients’ registration. The PI was required to furnish a written high-resolution computed tomography (HRCT) report from a radiologist trained in reading HRCT relevant to ILD. A written histopathology report was to be provided in cases where a biopsy was performed. The opinion of rheumatologists was also sought in cases of CTD-related ILD. The purpose of this multidisciplinary discussion (MDD) resource group was to maintain uniformity in the diagnostic criteria.

The questionnaire was specifically designed for the registry. After obtaining the written informed consent, patients more than or equal to 18 years of age with relevant history and HRCT images of the chest consistent with ILD were included. Data was gathered on demographics, occupation, history of environmental exposure, comorbids like diabetes, hypertension, chronic obstructive pulmonary disease (COPD), gastroesophageal reflux disease (GERD), history of drugs that can cause ILD like bleomycin, amiodarone, methotrexate, or cyclophosphamide and family history of ILD. Findings of HRCT scans, serology for CTD-ILD, and pulmonary function tests (PFTs), mainly consisting of spirometry and diffusing capacity of the lungs for carbon monoxide (DLCO) in a few cases, were recorded. Active lung parenchymal infections like pulmonary tuberculosis (TB) were ruled out by sputum or bronchoalveolar lavage (BAL) for acid-fast bacillus (AFB) smear, Gene-Xpert, and culture when there was a high clinical suspicion of infection. Patients with a clinical history of previous tuberculosis, lung carcinoma and history of exposure to radiation were excluded from the registry. The electronic copy of questionnaire was uploaded on an online database (http://pcsregistry.ildpak.com/public/login) while the physician retained a hard copy of the questionnaire as a source document.

Data was analyzed through Statistical Package for Social Sciences (SPSS) version-22 (IBM Corp, Armonk, NY, USA). Descriptive statistics was presented as mean±standard deviation for numerical data and frequencies percentages for categorical data. Analysis of variance (ANOVA) and Chi-square test of significance were applied to evaluate the association between type of ILD and study variables. P-value <0.05 was considered statistically significant.

## Results

A total of 744 patients were enrolled in the registry. The final ILD diagnoses were as follows: IPF 256 (34.4%), HP 132 (17.7%), iNSIP 125 (16.8%), CTD-ILD 121 (16.3%), sarcoidosis 68 (9.1%) and 42 (5.7%), and other ILD (Table 1).

**Table 1:** Relative Frequencies of Interstitial Lung Diseases in ILD Registry ILD=Interstitial Lung Disease, IPF=Idiopathic Pulmonary Fibrosis, HP=Hypersensitivity Pneumonitis, iNSIP=Idiopathic Nonspecific Interstitial Pnuemonitis, CTD-ILD=Connective Tissue Disease Associated ILD, CPFE=Combined Pulmonary Fibrosis with Emphysema, RA=Rheumatoid Arthritis, SD=Scleroderma, MCTD=Mixed Connective Tissue Disease, SLE=Systemic Lupus Erythematosus, PM/DM=Polymyositis/Dermatomyositis, SS=Sjögren's Syndrome, COP/BOOP=Cryptogenic Organizing Pneumonia/Bronchiolitis Obliterans Organizing Pneumonia, IPAF=Interstitial Pneumonia with Autoimmune Features, PAP=Pulmonary Alveolar Proteinosis, PLCH=Pulmonary Langerhans Cell Histiocytosis, LAM=Lymphangioleiomyomatosis, RB-ILD=Respiratory Bronchiolitis ILD, CWP= Coal Worker’s Pneumoconiosis, DIP=Desquamative Interstitial Pneumonia, AIP=Acute Interstitial Pneumonia, BO=Bronchiolitis Obliterans

Types of ILD	n (n%)
IPF	256 (34.4%)
CPFE	11 (1.5%)
HP	132 (17.7%)
iNSIP	125 (16.8%)
CTD-ILD	121 (16.3%)
RA	56 (7.5%)
SD	25 (3.4%)
MCTD	14 (1.9%)
SLE	13 (1.7%)
PM/DM	11 (1.5%)
SS	2 (0.3%)
Sarcoidosis	68 (9.1%)
Others	42 (5.7%)
COP/BOOP	15 (2%)
Drug Induced ILD	6 (0.8%)
Methotrexate	3 (0.4%)
Cyclophosphamide	2 (0.3%)
Interferon	1 (0.1%)
IPAF	5 (0.7%)
PAP	3 (0.4%)
PLCH	2 (0.3%)
LAM	2 (0.3%)
RB-ILD	2 (0.3%)
Silicosis	2 (0.3%)
CWP	1 (0.1%)
DIP	1 (0.1%)
AIP	1 (0.1%)
BO	1 (0.1%)
Undetermined ILD	1 (0.1%)

The median age was 57.5 years with interquartile range 46-65 years and gender was predominantly female (65.6%). Overall, 679 (91.3%) patients lived in urban areas. IPF patients were older (>60 years) and with history of current or past smoking with statistical significance (P<0.001). All ILD, except for IPF, had a statistically higher proportion of females (P<0.001). The most common clinical symptoms were cough in 674 (90.6%) and dyspnea in 519 (69.75%). On physical examination, crepitations were audible in 674 (90.6%) and rhonchi in 83 (11.2%) while clubbing was seen in 247 (33.2%). Gastroesophageal reflux disease (GERD) was present in 195 (26.2%) patients and was statistically significant with iNSIP and IPF (P=0.021). Echocardiography was done in 432 (58.1%) patients, of which 206 (47.7%) showed pulmonary arterial hypertension (PAH); 44.7% had mild PAH, 30.1% had moderate PAH and 25.2% had severe PAH (Table 2).

**Table 2:** Demographics and Clinical Features of ILD ILD=Interstitial Lung Disease, IPF=Idiopathic Pulmonary Fibrosis, UIP=Usual Interstitial Pneumonia, HP=Hypersensitivity Pneumonitis, iNSIP=Idiopathic Nonspecific Interstitial Pnuemonitis, CTD-ILD=Connective Tissue Disease Associated ILD, COPD=Chronic Obstructive Pulmonary Disease, GERD=Gastroesophageal Reflux Disease, PAH=Pulmonary Arterial Hypertension, mPAP=Mean Pulmonary Arterial Pressure

	Overall (n=744)	IPF/UIP (n= 256)	HP (n=132)	iNSIP (n=125)	CTD-ILD (n=121)	Sarcoidosis (n=68)	Others (n=42)	P-value
Age in years; mean ±SD	56.2 ±13.9	64.5 ±11.8	51.6 ±13.5	55.4 ±11.7	50.3 ±12.1	50.3 ±12.7	48.4 ±15.5	˂0.001
Gender; n (%)								˂0.001
Male	256 (34.4%)	139 (54.3%)	34 (25.8%)	23 (18.4%)	12 (9.9%)	23 (33.8%)	25 (59.5%)	
Female	488 (65.6%)	117 (45.7%)	98 (74.2%)	102 (81.6%)	109 (90.1%)	45 (66.2%)	17 (40.5%)	
Area of Living; n (%)								0.023
Rural	65 (8.7%)	32 (12.5%)	8 (6.1%)	15 (12%)	5 (4.1%)	3 (4.4%)	2 (4.8%)	
Urban	679 (91.3%)	224 (87.5%)	124 (93.9%)	110 (88%)	116 (95.9%)	65 (95.6%)	40 (95.2%)	
Smoking History; n (%)	113 (15.2%)	67 (26.2%)	9 (6.8%)	9 (7.2%)	8 (6.6%)	5 (7.4%)	15 (35.7%)	˂0.001
Clubbing; n (%)	247 (33.2%)	121 (47.3%)	43 (32.6%)	37 (29.6%)	30 (24.8%)	8 (11.8%)	8 (19%)	˂0.001
Hypertension; n (%)	288 (38.7%)	112 (43.8%)	40 (30.3%)	59 (47.2%)	46 (38%)	23 (33.8%)	8 (19%)	0.003
Diabetes Mellitus; n (%)	215 (28.9%)	72 (28.1%)	37 (28%)	53 (42.4%)	24 (19.8%)	21 (30.9%)	8 (19%)	0.003
COPD; n (%)	48 (6.5%)	30 (11.7%)	3 (2.3%)	6 (4.8%)	2 (1.7%)	3 (4.4%)	4 (9.5%)	0.001
GERD; n (%)	195 (26.2%)	77 (30.1%)	35 (26.5%)	39 (31.2%)	29 (24%)	8 (11.8%)	7 (16.7%)	0.021
Echocardiography; n (%)	432 (58.1%)	149 (58.2%)	86 (65.2%)	76 (60.8%)	78 (64.5%)	22 (32.4%)	21 (50%)	˂0.001
PAH (n=432); n (%)	206 (47.7%)	77 (51.7%)	39 (45.3%)	46 (60.5%)	29 (37.2%)	9 (40.9%)	6 (28.6%)	0.023
Severity of PAH (mPAP) (n=206); n (%)								0.508
Mild (25 – 40 mmHg)	92 (44.7%)	37 (48.1%)	17 (43.6%)	22 (47.8%)	9 (31%)	4 (44.4%)	3 (50%)	
Moderate (41 – 55 mmHg)	62 (30.1%)	19 (24.7%)	15 (38.5%)	13 (28.3%)	13 (44.8%)	1 (11.1%)	4 (44.4%)	
Severe (> 55 mmHg)	52 (25.2%)	21 (27.3%)	7 (17.9%)	11 (23.9%)	7 (24.1%)	4 (44.4%)	2 (33.3%)	

**Figure 2:**
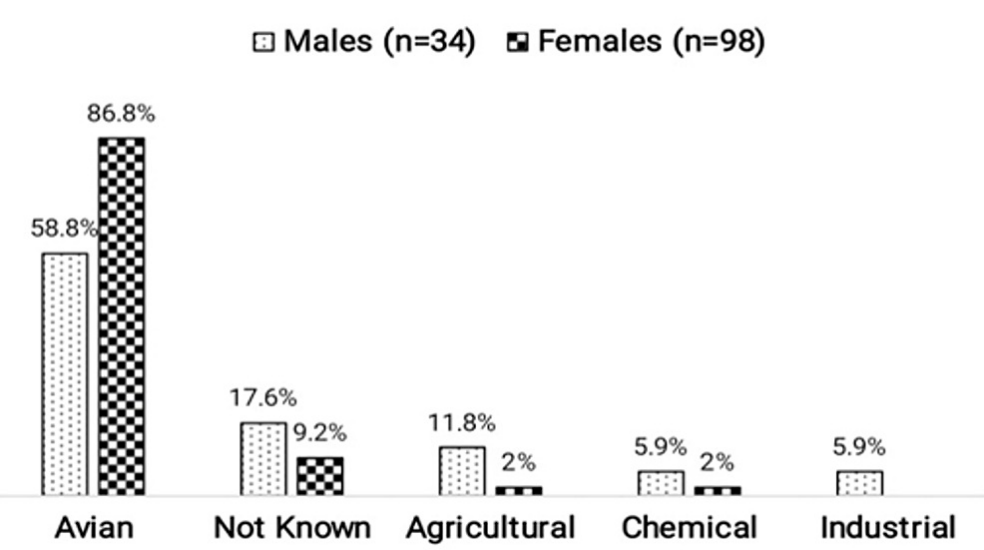
Gender-wise Exposure in Patients with Hypersensitivity Pneumonitis

**Figure 3:**
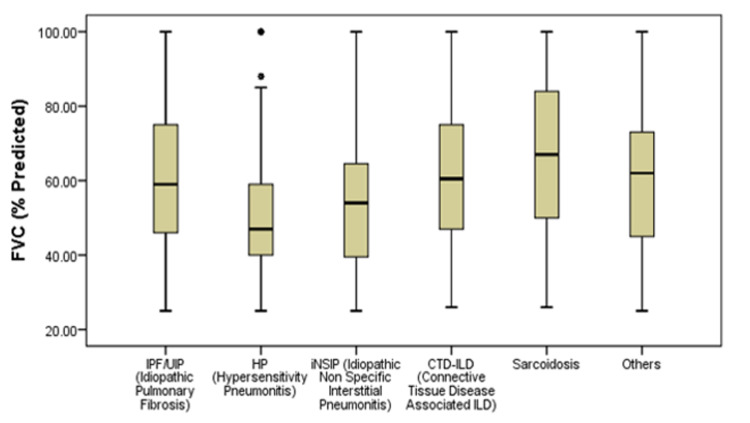
Box and Whisker Plot of Forced Vital Capacity (% of predicted) in Different ILDs FVC=Forced Vital Capacity, ILD=Interstitial Lung Disease

The overall predicted forced vital capacity (FVC) was 58.2 ±18.7%. FVC % of predicted was presented as box-and-whiskers plot showing the median, interquartile range and outliers (if any) for ILD patients. The median FVC for IPF was 59 (range: 25-99.99), for iNSIP was 54 (range: 25-99.99), for CTD-ILD was 60.5 (range: 26-99.99), for sarcoidosis it was 67 (range: 26-99.99). The HP patients had the lowest FVC % of predicted median that was 45.5 (range: 25-82) where four cases had ≥85 FVC and considered as outliers (Figure 2). An avian exposure was reported in 105 (79.5%) HP patients and out of which 86.8% were females (Figure 3).

## Discussion

IPF accounted for the highest proportion of reported ILD which is 34.4% in our registry. This finding is consistent with earlier local published data [8, 9]. IPF was diagnosed between 28.6% to 46% among ILD in various studies reported from India [11]. The ILD-India Registry however reported 13.7% IPF which is considerably lesser than their earlier data. Interestingly, the diagnosis of IPF (28.2%) among ILD was highest in their onsite reporting which was reduced to 13.7% by ILD experts from the US [4]. The data from a single-center study in Saudi Arabia reported IPF in 23.3% of patients while the Romanian, Turkish, and Greek registries showed a prevalence of IPF in 25%, 19.9%, and 19.5%, respectively [12-15]. In our registry, IPF was predominantly found in males and those greater than 65 years of age as reported worldwide [4, 12, 14-16].

The second most common ILD in our registry was HP (17.7%) whereas it accounted for the majority in the Indian registry (47.3%) [4]. Most other ILD registries have reported the prevalence of HP to be 3-13% [17, 18]. It has been gaining more proportion in our population from 4% to 17.7% in last 10 years. This could either be its true incidence due to an increase in environmental allergens or a better recognition of chronic fibrosing HP from IPF which is comparable to results from regional data [4-9]. In our population, 79.5% had an avian exposure which was more predominant in housewives reflecting significant avian antigen in the household environment. The data from the Japanese epidemiological survey also presented 60.4% while the Indian registry showed 21.4% of avian exposure [4, 19].

We have seen iNSIP (16.8%) and CTD-related ILD (16.3%) as the next two most frequent ILD in our registry. CTD-ILD was also the second common ILD in the Indian registry (13.9%) with rheumatoid arthritis (RA) being the commonest subtype followed by scleroderma which is similar to our findings [4]. Females were found to be significantly higher in iNSIP (81.6%) & CTD-ILD (90.1%) in comparison with other ILD. In CTD-ILD, females under the mean age of 50 years were predominant which is consistent with an international data [20].

Sarcoidosis was found in 9.1% which is much less than in earlier studies from Pakistan which reported 15.3% to 18.5% [5, 6, 9]. This difference could be attributed to misdiagnoses of sarcoidosis where it might be considered as a sequela of pulmonary tuberculosis in high TB burden countries [18].

There could be several limitations in our ILD Registry. The current data was collected from major urban cities of Pakistan which does not represent our rural population. The lack of use of a detailed questionnaire pertaining to the history of exposures and the non-availability of serum precipitins to identify inducers might have led to an underdiagnosis of HP. Differentiating fibrotic HP from IPF has generally remained a difficult task as well.

Another limitation in our registry was the scarcity of histopathological confirmation attributable to a number of factors. The majority of cases were IPF which do not require lung biopsy while the iNSIP and HP presented with severely restricted lung function.

## Conclusions

Registry data from Pakistan concludes that IPF was the most common ILD followed by HP, iNSIP, CTD related ILD and sarcoidosis. There has been slight variation in the relative frequencies obtained from various geographic areas of this populous country, but most values have been in line with earlier studies. An expansion of the ILD registry to other cities and rural areas of the country with longitudinal follow-up of currently enrolled patients and more stringent validation of data by MDD in tertiary care and National Coordinating Centers could further expand the network and refine disease information.
